# Accessibility to prenatal care at the Street Outreach Office: nurse perceptions in northern Brazil

**DOI:** 10.1590/0034-7167-2024-0090

**Published:** 2024-08-30

**Authors:** Nayara Gonçalves Barbosa, Karoline Cardoso Netto, Lise Maria Carvalho Mendes, Thaís de Oliveira Gozzo, Herla Maria Furtado Jorge, Andyara do Carmo Pinto Coelho Paiva, Thaís Vasconcelos Amorim, Flávia Azevedo Gomes-Sponholz

**Affiliations:** I Universidade Federal de Juiz de Fora. Juiz de Fora, Minas Gerais, Brazil; II Universidade de São Paulo. São Paulo, São Paulo, Brazil; III Universidade Federal do Amapá. Macapá, Brazil; IV Universidade de São Paulo. Ribeirão Preto, São Paulo, Brazil; V Universidade Federal do Piauí. Teresina, Piauí, Brazil

**Keywords:** Homeless Persons, Women’s Health, Prenatal Care, Health Services Accessibility, Nurses, Personas con Mala Vivienda, Salud de la Mujer, Atención Prenatal, Accesibilidad a los Servicios de Salud, Enfermeras y Enfermeros

## Abstract

**Objectives:**

to understand nurse perspectives regarding homeless pregnant women’s accessibility to prenatal care.

**Methods:**

a qualitative study, with analysis based on the concept of accessibility. Semi-structured interviews were carried out with 11 nurses who work at the Street Outreach Office in northern Brazil.

**Results:**

nurses are faced with geographic barriers and dangerous situations in border regions, recognizing that there is a context of physical, sexual and psychological violence that involves homeless pregnant women who seek care at the Street Outreach Office. Street Outreach Office nurses’ work occurs in conjunction with other services in the Health Care Network. The implementation of educational measures is a powerful strategy, as is establishing links with women.

**Final Considerations:**

the Street Outreach Office’s work provides meetings with pregnant women on site in the territory, which can provide geographic and socio-organizational accessibility to prenatal care.

## RESUMEN

**Objetivos:**

comprender la perspectiva de enfermeros sobre la accesibilidad de las mujeres embarazadas en situación de calle a la atención prenatal.

**Métodos:**

estudio cualitativo, con análisis basado en el concepto de accesibilidad. Se realizaron entrevistas semiestructuradas con 11 enfermeros que trabajan en Consultorio en la Calle en el Norte de Brasil.

**Resultados:**

los enfermeros enfrentan barreras geográficas y situaciones peligrosas en regiones fronterizas, reconociendo que existe un contexto de violencia física, sexual y psicológica que involucra a mujeres embarazadas en situación de calle que acuden a atención en Consultorio en la Calle. El trabajo de los enfermeros en Consultorio en la Calle se da en conjunto con otros servicios de la Red de Atención a la Salud. La implementación de medidas educativas es una estrategia poderosa, al igual que establecer vínculos con las mujeres.

**Consideraciones Finales:**

las actividades de Consultorio en la Calle brindan encuentros con mujeres embarazadas in situ en el territorio, lo que puede brindar accesibilidad geográfica y socioorganizacional a la atención prenatal.

## INTRODUCTION

Access to prenatal care is a woman’s right in Brazil, and accessibility to quality maternal prenatal care can prevent the occurrence of preventable maternal deaths^(1)^. The United Nations (UN) and the World Health Organization (WHO) have sought to reduce this indicator by defining global commitments, such as the Sustainable Development Goals (SDG)^(2)^. SDG target 3.1 regarding the reduction of the maternal mortality ratio (MMR) established reducing the global MMR to less than 70 maternal deaths per 100,000 live births by 2030^(2)^.

One of the strategies suggested for reducing MMR is prenatal care^(1)^. However, access to health services and prenatal care is limited for many pregnant women, especially those who are in vulnerable situations, such as those belonging to ethnic minorities, in situations of poverty, homeless, migrants and refugees, sex workers, women exposed to domestic violence, people with mental health problems, substance users, among others^(3-4)^. Such inequities in accessibility to health services among women reflect the profile of maternal mortality, with a predominance of black women, of low socioeconomic status and with low education^(3)^.

Homeless people are defined as those who live in conditions of extreme poverty, who have broken family ties, who do not have conventional housing and occupy public spaces to live and seek forms of support, temporarily or permanently, in addition to using overnight accommodation^(5)^. Women accounted for approximately 18% of the homeless population in Brazil, and a significant portion of these are concentrated in the age group of 18 to 55 years, many of them in the reproductive period^(5)^. Precarious social and health conditions favor unintended pregnancies, demonstrating flaws in reproductive planning actions, in addition to contributing to unsafe abortions, increasing the risk of maternal morbidity and mortality^(6-8)^.

In the Brazilian context, the intergenerational configuration of homeless families is evident^(9)^, contributing to the perpetuation of the poverty and misery cycle. Furthermore, barriers such as prejudice and stigma produced, lack of financial resources, lack of mobility and lack of programs aimed at pregnant women living on the streets highlight the difficulty in accessing women to undergo prenatal care^(9)^, representing risk factors for pregnant woman’s fetus’ health^(4,7-8,10)^. The effect of alcohol, tobacco, use of illicit substances, low nutritional intake, maternal comorbidities, exposure to pollution and environmental factors, in addition to street violence and unsafe sexual practices as aggravating factors for pregnant woman’s fetus’ health, must be considered^(4,7,10-12)^.

Faced with such challenges, the Street Outreach Office team seeks to coordinate and provide comprehensive health assistance to the homeless population in the territory, with the incorporation of disease prevention and health promotion practices and expanding access to care networks, in addition to ensuring the rights of this population^(13)^. The Street Outreach Office operates on an itinerant basis, developing on-site actions in the streets’ living territory, building its therapeutic setting based on the bond and welcoming of the homeless population according to the territory’s specificities, context and characteristics^(13-14)^. The Street Outreach Office is in line with SDG 3, which seeks to ensure healthy lives and promote well-being for all, leaving no one behind^(2)^.

Nursing work reflects care for individuals, families and the community, with the performance of care, management, educational and investigative activities to promote, maintain and recover individual and collective health^(15)^. In this direction, the role of nurses stands out, as Street Outreach Office team members, to contribute to homeless pregnant women’s accessibility to prenatal care, taking into account their vulnerabilities and different care needs in relation to the general population.

In this context, it is essential to study nurses’ work process in prenatal care within the scope of the Street Outreach Office to better understand their role in caring for homeless women during the pregnancy-puerperal period. Carrying out such studies has the potential to identify nurses’ understanding of the object, the instruments used, the purpose and the final product obtained with the actions developed by this professional^(15)^. Considering the above, the present study seeks to encourage reflections considering nurses’ daily work with pregnant women living on the streets in the Street Outreach Office, contributing to achievement the SDG and directing public policies for this population.

## OBJECTIVES

To understand nurse perspectives regarding homeless pregnant women’s accessibility to prenatal care.

## METHODS

### Ethical aspects

The study followed the ethical precepts specified in Resolution 466 of December 12, 2012 of the Brazilian National Health Council for research carried out with human beings. The research was approved by a Research Ethics Committee linked to the Brazilian National Research Ethics Commission (CONEP - *Comissão Nacional de Ética em Pesquisa*) in May 2022.

### Study design and setting

This is research with a qualitative approach^(16)^. The scope of research was guided by COnsolidated criteria for REporting Qualitative research (COREQ)^(17)^ guidelines. The study was based on the conceptual bases of accessibility proposed by Donabedian^(18)^. Accessibility is understood as the ability to produce offers and respond to the population’s needs, considering the characteristics of health services and resources that contribute to or represent obstacles to their use by users^(18)^. This author distinguishes two dimensions of accessibility, with socio-organizational accessibility referring to the characteristics of the resources offered and geographic accessibility involving the characteristics related to the distance and time necessary to reach and obtain the services^(18)^.

The study was carried out in Street Outreach Office services in the states of Acre, Amapá, Amazonas, Pará and Tocantins, located in northern Brazil. These services serve the homeless population, sex workers, migrants, *Warao* indigenous people and other vulnerable populations in the territory. According to the number and professional composition, Street Outreach Office teams are classified into modalities I, II and III. In modality I, the team is made up of four professionals, two with higher education and two with technical level. In modality II, there are six professionals, three with higher education and three with technical level. Modality III has the same constitution as II, plus a medical professional^(19)^. In the present study, Street Outreach Office teams from the three modalities were included, composed of professionals such as nurses, doctors, psychologists, social workers, nutritionists, physical educators, nursing technicians, oral health technicians and social workers.

### Population and selection criteria

The reference population consisted of nurses who worked in Street Outreach Office teams in northern Brazil. Nurses who had been working at the Street Outreach Office for at least six months and who provided prenatal care were included. The sample was obtained through the convenience sampling process. Initially, the state and municipal health departments were asked to contact Street Outreach Office coordinators so that, via email, the research could be presented to them and the intermediation of researchers’ access to Street Outreach Office nurses, potential participants in this research, could be arranged. Subsequently, emails were sent to all nurses who met the inclusion criteria. Data collection took place between October 2022 and January 2024.

### Data collection

A pilot study was carried out with the aim of identifying difficulties and improving instrument application, and there was no need for modifications. The awareness-raising interviews were conducted by one of the authors, a nurse, with a doctoral degree and experience in qualitative research.

Data were obtained between October 2022 and January 2024 through a semi-structured interview, carried out remotely and recorded on the Google Meet^®^ videoconferencing platform. After expressing interest and agreement to participate in the study, by signing the Informed Consent Form, one of the researchers contacted a nurse to schedule the interview. The interviews were carried out by two nurse researchers, with doctoral and master’s degrees, with experience in qualitative research, and a nursing academic qualified for the role. The interviews lasted an average of 40 minutes and were carried out once per participant.

The interviewers had no prior contact with services and/or participants. The number of participants was defined using the data saturation criterion, with the inclusion of 11 nurses. The data saturation criterion involved the researcher’s perception during the continuous process of data analysis. Given the repetitions of speeches and the absence of new statements, the interviews were finalized^(20)^.

The data collection instrument followed a semi-structured script composed of two parts: the first composed of closed-ended questions, referring to participant sociodemographic characterization, including age, self-reported color, level of training (specialization, master’s degree, doctoral degree), time working at a Street Outreach Office; and the second composed of open-ended questions related to nurses’ care for homeless women. The guiding questions were asked: how is the approach to pregnant women in the Street Outreach Office? How do nurses provide prenatal care to homeless women?

After data collection, the interviews were transcribed. To guarantee participant secrecy and confidentiality, the interviews were identified using the letter “N”, for nurse, followed by an ordinal number, according to the order in which the interviews were carried out.

### Data analysis

The data were presented based on inductive thematic analysis^(21)^, based on six stages, which are: 1) familiarization with data; 2) generation of initial codes; 3) topic search; 4) topic review; 5) topic definition and naming; 6) report production and analyzed in light of the concept of accessibility (Chart 1).

**Chart 1:** Research data coding, Macapá, Amapá, Brazil, 2024

Initial codes	Intermediate codes	Final codes
On-site performance	Geographic context and security	Challenges in homeless pregnant women’s accessibility to prenatal care
Barriers to geographic accessibility		
Issues of security and dangerousness of the territory		
Migration of women in the territory		
Particularities of border regions	Migration and refugees in situations of social vulnerability	
Migratory movements of immigrants and refugees		
Context of vulnerability, late identification of pregnant women	Social vulnerability and health	The influence of homeless pregnant women’s living context on accessibility to prenatal care
Dependence on alcohol and other drugs		
Scarce food		
Prostitution, transactional sex		
Physical, sexual, psychological, symbolic and state violence	Violence and abuse	
Discontinuation of pregnancy, unfavorable obstetric outcomes	Sexual and reproductive health	
Limited provision of social resources	Access to social resources and health services	
Challenges in integrating the Health Care Network		
Offering a greater number of appointments at shorter intervals	Accessibility and quality of prenatal care	The potential of nurses’ work in prenatal care for homeless pregnant women’s accessibility
Accessibility to prenatal care		
Raising awareness of pregnant women’s rights	Maternal and neonatal health promotion	
Risk approach		
Harm reduction		
Creating links	Developing therapeutic relationships	

## RESULTS

Of the 11 participating nurses, eight were women and three were men, with a mean age of 35 years. Furthermore, six identified themselves as mixed race and six were married/in a stable relationship. The mean training time was nine years; nine had specialization; and two had a master’s degree. The mean time working at the Street Outreach Office was six years, with contracts or public tenders. Most Street Outreach Office services had mobile service units, such as vans, and only one service had a team transport car.

### Challenges in homeless pregnant women’s accessibility to prenatal care

The organizational dimension of Street Outreach Office team nurses’ work has the potential to reach pregnant women experiencing homelessness through on-site work to promote accessibility to prenatal care. To this end, nurses’ strategy is to use the territory’s social equipment to care for these women, highlighting Basic Health Units (BHU), linking them to the Health Care Network.

*We go on the route and try to bring them, because their difficulty is getting to BHU. Because, sometimes, it’s far away, things like that, and we try to bring her in the car from the Street Outreach Office to the BHU to do this service, go there, right? Often, there is a Family Strategy team that works close to where they stay*. (N4)

*We work a lot as a team, but I do prenatal care on the street. As we don’t have a van* [mobile unit], *when I feel the need to carry out an assessment, such as palpation, uterine height, we go to the Family Health Unit closest to the territory where it is located to carry out a specific assessment, rapid testing, vaccination.* (N5)

However, there are barriers in geographic accessibility due to women moving to other territories. It is also worth highlighting the dangerous nature of certain regions, which prevents the Street Outreach Office team from entering for security reasons, making it difficult for professionals to meet pregnant women for prenatal care.

*We do the initial exams and send them to the Basic Health Unit, and we monitor them* [...] *but they end up disappearing. Over the course of these nine months, they change territories, and then, when we go to see, the prenatal period has already ended and she appears with her baby on her lap.* (N3)

*They do not have prenatal care. I have a patient who I have been with for two months, wanting to bring her for prenatal care. And every time I go on the route, I can’t find her, and then it becomes difficult for us to follow up. I should already be taking ferrous sulfate and folic acid.* (N4)

*Depending on where they are, there are some that we don’t go into, for team safety. So, we really deal with their interest, with their availability of wanting. It is a task to make them aware of the importance of having prenatal care.* (N1)

It is also noteworthy that the border regions were considered challenging areas for care, which receive the impacts of social phenomena related to the migratory movements of immigrants and refugees, with emphasis on *Warao* indigenous people from Venezuela, in an intensified manner.

*It’s complicated, especially for those in the border area, because they end up receiving and dealing with a lot of information. The vast majority of them are indigenous, so when they come from Venezuela, they are fleeing the issue* [of the economic crisis] *in the country. The municipality is immersed in a major crisis, because they come from the forest. They are indigenous, they will do what they call on the street “hose”, which is begging. They are on the street, others manage to go to a place to stay in a shelter, but they spend the whole day living like the homeless population here.* (N1)

*Those who suffer the impacts* [of immigration] *first are those at the border. Most of the immigrants on the street come from Venezuela, they are indigenous, and registration is very complicated as well as entering the territory.* (N3)

Prenatal care provided by nurses to homeless women often does not follow the schedule recommended by the Ministry of Health, due to the late identification of pregnancy by women. The late recruitment of pregnant women, when in the third trimester, directs the first care to secondary and tertiary care points, limiting themselves to hard technologies that can detect greater or lesser risks.

*I scheduled their prenatal care at BHU, I did an active search, none of them were picked up early, they were from 20 weeks onwards.* (N10)

*For women, when they are already pregnant, we try to provide them with prenatal care, but generally we already find out about the pregnancy when they are almost ready to give* [birth]. *So, generally, prenatal care does not exist; prenatal care is straight away to go to the maternity ward.* (N1)

[...] *there are some that we will only find out about pregnancy at the end of pregnancy. So, we are already trying to take her to the hospital* [high-risk monitoring]. *On the day we meet, we do an ultrasound, see what week it is, do quick tests to find out if there is any disease, we do what we can.* (N2)

### The influence of homeless pregnant women’s living context on accessibility to prenatal care

The recognition, by professionals, that homeless women are inserted in a context of physical, sexual, psychological, symbolic and state violence, favoring dependence on alcohol, tobacco and other illicit drugs, with scarce food, prostitution, subsistence sex, among other specificities, is fundamental to articulate nursing care and socio-organizational accessibility.

*Women are already very exposed, but these women who live on the streets are even more exposed, because they suffer violence, many of them suffer sexual violence.* [...] *they stay in the square or some commercial location alone, and they end up using alcohol and other drugs while still pregnant. The women I assist, most of them are alcohol users, and we try to guide them during this period.* (N4)

*Many women bring the desire to become pregnant, a health need that I identify as a nurse. Women who suffer multiple violence on the streets, both sexual, physical and moral, including from public workers.* (N5)

*We are pregnant with twins, and we are following this prenatal care. She is still homeless, she is sleeping on the street, and then she continues to use drugs, continues to prostitute herself.* (N6)

*The majority are drug users and, even during pregnancy, they continue using drugs and drinking.* (N10)

Pregnancy interruption was mentioned by nurses as frequent and the causes as being difficult to identify. The context of life on the streets can favor the outcome of abortion, whether due to women’s precarious health conditions or the violence they are exposed to on the streets.

*Regarding abortion, we have many reports from women* [...] *the complicated thing is that we don’t really know* [how it happened], *as they only report it later, we don’t know if it was fetal alcohol syndrome, we can’t say if it was mistreatment, if it was violence, what it actually was, trying to identify the cause. Most of us have stories from the women themselves.* (N7)

*The patient told me that she got involved in a fight with another woman, and then she was kicked in the stomach. She was already eight months old and lost the baby.* (N6)

*Then we took* [pregnancy test], *it was positive. When it came to non-acceptance, she said, “I don’t accept it because I can’t have it”. That whole thing and I believe she was trying to abort, I don’t know how, she arrived the following week complaining of abdominal pain and bleeding and we, as a team, took her to the referral hospital.* (N3)

Furthermore, the limited provision of social resources for homeless pregnant women and the lack of Health Care Network integration for humanized care for homeless women in the pregnancy-puerperal cycle stand out.

*One of our challenges with homeless women is in relation to the provision of general care, but mainly sexuality and reproduction. They have the desire to become pregnant and, unfortunately, when we talk about networks* [in the municipality), *we do not have any support for welcoming homeless people. We don’t have a hostel, we don’t have a pop center, we don’t have a halfway house. And this is where we come into the harm reduction perspective. Telling her that because she is in a vulnerable context, she is already putting herself at risk, even more so, right?* (N5)

*I was upset when I arrived at the maternity ward, because she gave birth alone. They didn’t let her partner in. There is no such thing as just because she is homeless, does she have to give birth alone?* (N10)

### The potential of nurses’ work in prenatal care for homeless pregnant women’s accessibility

In prenatal care, the role of nurses in making pregnant women aware of their rights to have access to health services and to be welcomed and cared for with dignity and respect stands out.

*It is the issue of guaranteeing rights. They understand that they have rights and they can empower themselves. We try very hard to empower them: that they have the right to have prenatal care at the health unit, just because I’m giving her prenatal care on the street doesn’t mean she doesn’t have the right to go to the health unit.* (N7)

Given the complex health needs of pregnant women living on the streets, the Street Outreach Office’s work to provide accessibility to prenatal care occurs jointly and in coordination with the different services that make up the Health Care Network, with the development of integrated and shared actions with BHU, Family Health Strategies (FHS), Psychosocial Care Centers (CAPS - *Centros de Atenção Psicossocial*), emergency services and other levels of care, according to women’s needs. One strategy used by nurses was to provide a greater number of appointments, at shorter intervals, to monitor pregnant women.

*Prenatal care happens in a very clear way, it follows standards, even though it is at the Street Outreach Office. We do any and all tests, we schedule an ultrasound, we have the vitamins that this woman has to take throughout these nine months and, if it is a normal prenatal care, let’s say,* [will be monitored] *in the Family Health Strategy. This woman goes once to the doctor, once to the nurse.* [...] *because I am a vulnerable woman, I make this return in a short space of time, then I can have better, closer monitoring with her. She will always be there, in my eyes*. [...] *we take them to the reference hospital, guaranteeing this care.* (N6)

[...] *here, we take this care so that she does not remain homeless throughout her pregnancy: she is hospitalized at CAPS AD* [Center for Psychosocial Care for Alcohol and Drugs] *if she is a user of alcohol and drugs. And the two* [pregnant women] *we had, unfortunately they were dependent.* (N8)

*When they continue to the end with us, they are accompanied to the hospital, to a maternity ward for high-risk births. They end up being classified as high risk, due to being homeless and not having proper prenatal care, staying well alternated: appointments, exams, vitamins, vaccines, it gets pretty messy, and then we can forward them and monitor them postpartum and provide the follow-up that is necessary for them.* (N3)

The implementation of educational measures is a powerful strategy for nursing care and promoting socio-organizational accessibility during prenatal care for homeless pregnant women. Addressing the risks of certain harmful practices during pregnancy has the potential to contribute to raising awareness and changing habits, with a view to reducing damage to the dyad’s health.

*I talk to them a lot about the problems that she can acquire both during pregnancy and when the child is born, because she is using these substances. We talk to them a lot.* (N4)

*It is a gradual achievement for her* [user monitored at the Street Outreach Office], *who is becoming aware of her pregnancy. She reports that she is using drugs in smaller quantities. So, it is an achievement for the Street Outreach Office to know that we are transforming small realities that will generate a big impact on their lives. For me, it’s one of the best things to know that we can transform, because knowledge is transformative.* (N7)

The creation of bonds between nurses and women is noted as a fundamental element of care, with repercussions on their adherence to prenatal care.

*In fact, I have a patient, a patient who is so difficult, because she is so difficult, that we like her. She is passionate because she is so difficult. She is on her second child. In the first one, she did all the prenatal care at the Street Outreach Office. She is a patient that I am particularly moved by because she created a great bond with the team.* [...] *she is on her fifth appointment. She is 28 weeks, and we will be able to do more than six appointments. We were able to do a quick test, we were able to “supplement her”, we were able to provide a lot of guidance.* (N7)

From this perspective, the role of nurses in prenatal care for homeless pregnant women has the potential to promote access to the health system and provide satisfactory results within women’s possible conditions.

*She arrived with a very characteristic low weight; she is gaining weight. So, we are seeing how much she is improving, how much better she feels, how much she seeks prenatal appointments, she talks to us on the street, “I have a few days left until my appointment!”. Knowing that the person wants to receive an appointment is the most rewarding thing, because normally we remain in this identification, in this active search. The fact that she seeks care and it is within the normal range, within the month, within the day of appointment, as recommended monthly, every 15 days, once a week and she manages to achieve this, is a very good.* (N7)

## DISCUSSION

The conditions of homeless pregnant women violate their human, sexual and reproductive rights and their rights to a life of dignity. Homeless pregnancy refers to a condition of risk and social vulnerability, a context of precarious life, rupture of affective and social bonds, and an inadequate space for gestation^(8)^. It is important to highlight the exposure of women to situations of street violence, sexual abuse, exposure to sexually transmitted infections (STI), substance abuse, rejection by families due to pregnancy, shame, sadness, dissatisfaction, guilt over unintended pregnancy and uncertainty about the future^(7-9,13,22)^.

The present study identified that on-site Street Outreach Office nurses’ work contributed to the socio-organizational and geographic accessibility of homeless pregnant women, often constituting the gateway to health services for prenatal care. Actions on the street and active search represent essential devices to guarantee access to care^(14)^.

Practice in transit mobilizes the meeting between different workers and travels through health and intersectoral networks, seeking articulation to care for people who were invisible in the Brazilian Health System scenarios^(14,23)^.

When considering the risks of a homeless pregnancy, the need for integration between Primary Health Care services as well as care from a specialized and multidisciplinary health team, in secondary or tertiary reference services stands out^(22,24)^. However, the articulation between Street Outreach Office and services that make up the Health Care Network and the intersectoral network may be fragile, considering the difficulties of services in meeting the more complex demands of homeless people and the absence of formalized flows between the territory’s equipment^(14)^.

Late onset, difficulties in follow-up, or lack of prenatal care were realities reported by nurses, given that accessibility, such as provision of care, can help in understanding variations in the use of health services by population groups^(18)^. The difficulty of pregnant women living on the streets in having their first contact with a professional, starting and/or continuing prenatal care was identified, in addition to women’s lack of knowledge about where to seek services^(4)^. Additionally, the low connection of the homeless population with Primary Health Care services means that the gateway to health services is predominantly through emergency care, with gaps in continuity of care^(14,25)^.

Barriers to homeless pregnant women’s accessibility to health services may be related to the health system structure and inadequacy to the needs and specificities of this group^(7,14)^, also considering the adequacy of professionals and resources used to the assistance of this group^(18)^. Furthermore, accessibility, considering the location of services and lack of transport, is part of the reality of people living on the streets^(7)^. Pregnant women in this situation usually need help from other people to find transportation or need to walk long distances to prenatal appointments, which results in absenteeism^(7)^. Nurses’ reports showed that the Street Outreach Office can use its own devices to transport homeless pregnant women, when available, as well as devices from the Health Care Network and social equipment in the territory, with emphasis on BHU, to promote accessibility to prenatal care for these pregnant women.

It is also noteworthy that border regions were described as scenarios weakened by the growing migratory movement to Brazil, with the need to adapt to the immediate demand imposed on them, often resulting in health system overload^(26)^. Venezuela is experiencing a political, economic and social crisis, and 33 Venezuelans, on average, enter Brazil every hour, approximately 800 per day. Among Venezuelan immigrant women, a very significant group that migrated to northern Brazil, especially to the capitals of Manaus, Belém and Boa Vista, are the *Warao* indigenous women^(27)^.

Immigrant pregnant women are less likely to receive adequate prenatal care, and there is an increased risk of adverse outcomes in the pregnancy and postpartum period^(28)^. In this context, the cultural competence of healthcare professionals plays a fundamental role in ensuring effective and culturally sensitive healthcare. However, it is important to recognize that cultural competence goes beyond superficial knowledge of refugees’ traditions and customs, as it involves a deep understanding of their unique experiences, needs and perspectives as well as recognition and respect for their cultural diversity. Furthermore, it is essential to consider the impact of trauma and previous healthcare experiences on refugees, which may influence their attitudes and behaviors towards healthcare services^(26)^. It is extremely urgent and important to carry out training to familiarize health professionals with immigrants’ language and customs, especially Venezuelans, in order to promote transcultural care that encompasses individuals’ diversity^(29)^, with a view to achieving accessibility and universality of services and respecting the specificities of traditional medicine practiced by these people.

The tensions and lack of trust of users in health professionals, as these often present problems in court^(14)^, in addition to inferior treatment, prejudice, stigma, exclusion, language and cultural barriers and fear can corroborate dissatisfaction, fragility of care, delay in seeking and continuing prenatal care for homeless pregnant women, worsening conditions of vulnerability^(4,7-8)^. Furthermore, previous negative experiences and the fear of losing custody of children can influence women not to seek health services during pregnancy^(7)^. Thus, it must be considered that health care for homeless pregnant women does not only refer to individual factors, but to the entire political-socioeconomic system in which health services are inserted^(7,25)^.

Due to the complexity involved in homelessness, it is essential to consider that the needs of this population are different and, consequently, require different resources/methods for their care^(25)^. In this regard, users may need care technologies that are not always common in the routines of health professionals, which go beyond protocolized conduct that tends to standardize care offerings^(23)^. It is noteworthy that homeless pregnant women experience fragility in person-centered care and the difficulty of geographic accessibility to health services, in addition to the lack of individualized care, considering that the needs and singularities of this population remain a challenge for health systems^(7)^. The way services are organized to respond to the challenges posed by women has the potential to impact better gestational outcomes beyond the limits of biomedical and care practices, guaranteeing the rights of women and their children^(30)^.

It was necessary to offer legal means of assistance by public authorities, with spaces and actions that can reduce stigma, pain, suffering and social exclusion^(8)^. Furthermore, it was necessary to develop strategies to combat the condition of being pregnant on the streets as well as to promote care that values these women’s ways of existing, with the implementation of health practices and procedures that promote health promotion, disease prevention, early diagnosis^(8)^, and strengthening the social network and support for pregnant women^(7)^.

In the present study, it was identified that a low level of knowledge and health literacy are detrimental factors for prenatal and postnatal care of homeless women. Important limitations regarding resources for obtaining information stand out, with many women unaware of the risks of using alcohol and substances for the growth and development of fetuses^(7)^. From this perspective, the health education strategies carried out by Street Outreach Office nurses contribute to these pregnant women’s access to information about health conditions, risks and injury prevention.

Concomitant to the guidelines on care during pregnancy, it is essential to link to the place of birth, guarantee qualified access to that place and to a humanized birth, care for women in the postpartum period and newborns, with offering postpartum reproductive planning, in addition to necessary intersectoral articulations according to demands, such as monitoring social assistance services, receiving benefits or income transfers, insertion in housing programs, among others^(31)^.

Vulnerability conditions concerning homeless women are expressed in several health needs^(11)^. Experiencing motherhood in this context is extremely complex, marked by profound unequal relations of class, race and gender^(7,30)^. A context of doubt prevails between the defense of women’s rights and the defense of fetuses/children. Criminalization and judgments regarding the inability of homeless women to exercise motherhood represent a trend in which fetus/child protection implies the retraction of women’s rights. During prenatal care, the use of legal and normative devices focused on punishment generates distrust, leading to abandonment and women’s refusal to seek social assistance and health services^(30)^. Although streets’ condition is far from the ideal for the exercise of motherhood, women’s desire to mother and the need for public policies that support women, families and children anchored in human rights perspective must be considered^(30)^.

It is considered that the potential solution to reduce the rates of unintended pregnancies in homeless women would be access to reproductive planning actions, which includes counseling. This is an action by nurses, in which information is offered in understandable language, especially in relation to effective methods of contraception, including long-lasting reversible ones, and ways to access contraceptive methods, if women wish to do so^(32)^. Recommendations for reproductive counseling and planning can be provided during prenatal care and also in the immediate postpartum period^(33)^.

To guarantee accessibility to health services, it is necessary to train professionals to welcome homeless women^(8)^, knowledge about the Brazilian National Policy for the Homeless Population and awareness about the rights of these users, since universal access is guaranteed in the Federal Constitution^(13-14,25)^. It is clear, given the results of this study, SDG 1 - No poverty, SDG 3 - Good health and well-being and SDG 5 - Gender equality still require public policy efforts to be achieved in Brazil.

Prenatal care provided by the Street Outreach Office can help achieve some SDG. SDG 1 mentions ending poverty in all its forms, everywhere. Regarding this aspect, it is known that the relationship between poverty and health is well established in the literature^(34)^. Currently, northern Brazil shows significantly higher levels of poverty than in the rest of the country^(35)^. Therefore, proposed targets with special attention to the poorest and most vulnerable populations and ensuring that they have access to basic services, such as prenatal care, are fundamental.

SDG-3 implies ensuring a healthy life and promoting well-being for all, at all ages. SDG-5 mentions achieving gender equality and the empowerment of all women and girls. In the North region, indicators such as maternal and infant mortality rates, as well as others, present worse conditions than in the rest of the country^(33)^ and, therefore, the goals related to these objectives are directly related to health conditions in the Amazon region. Proposals such as reducing preventable deaths of mothers, newborns and children under five by 2030, ensuring prenatal appointments, guidance on contraceptive methods, sexual and reproductive rights are fundamental for a healthy life and women’s and girls’ well-being, especially among those who are in a more vulnerable situation, such as homeless women.

### Study limitations

The limitations of this study refer to the interviews being carried out remotely and the impossibility of observing the care provided by the Street Outreach Office team, especially nurses. Although data collection in the virtual environment may pose risks, measures were taken to protect, security and preserve the rights of research participants^(36)^. The main researcher downloaded the data, deleting all records in shared virtual environments. It should be noted that carrying out the remote study enabled the inclusion of nurses from different northern states and the knowledge of different realities in prenatal care for pregnant women living on the streets.

### Contributions to nursing, health, or public policy

This study highlighted the importance of nurses’ actions, as Street Outreach Office team members, for geographic and socio-organizational accessibility to prenatal care for homeless pregnant women and coping strategies. The role of nurses in prenatal care at the Street Outreach Office contributes to homeless pregnant women’s accessibility to prenatal care, being important and supportive in favor of the dyad’s health. It is also important for implementing public policies, contributing to health and well-being, reducing maternal and child mortality, tackling social inequalities and defending women’s human rights as essential elements for achieving the SDG. Nursing care at the Street Outreach Office proved to be a powerful strategy for strengthening bonds, welcoming and tracking the health needs of homeless women.

## FINAL CONSIDERATIONS

Prenatal care by the Street Outreach Office nursing team is full of challenges, as can be seen with the occurrence of late enrollment, discontinuity of monitoring, changes of territory and precarious living conditions inherent to living without housing. However, the Street Outreach Office’s work provides meetings with pregnant women on-site in the territory, providing geographic and socio-organizational accessibility to prenatal care, with welcoming, building bonds, health education and inclusion in the Health Care Network From this perspective, Street Outreach Office nurses contribute to ensuring the right to health of homeless pregnant women, contributing to achievement the SDG.
