# Supplementary material for: Accessibility to prenatal care at the Street Outreach Office: nurse perceptions in northern Brazil
Source: Rev Bras Enferm. 2024 Aug 30;77(Suppl 2):e20240090. doi: 10.1590/0034-7167-2024-0090 (PMC11370769; doi:10.1590/0034-7167-2024-0090)
Supplement: Translation

# Translation: Acessibilidade ao cuidado pré-natal no Consultório na Rua: perspectivas de enfermeiros da região Norte do Brasil

**DOI:** 10.1590/0034-7167-2024-0090pt

**Authors:** Nayara Gonçalves Barbosa, Karoline Cardoso Netto, Lise Maria Carvalho Mendes, Thaís de Oliveira Gozzo, Herla Maria Furtado Jorge, Andyara do Carmo Pinto Coelho Paiva, Thaís Vasconcelos Amorim, Flávia Azevedo Gomes-Sponholz

**Affiliations:** I Universidade Federal de Juiz de Fora. Juiz de Fora, Minas Gerais, Brasil; II Universidade de São Paulo. São Paulo, São Paulo, Brasil; III Universidade Federal do Amapá. Macapá, Brasil; IV Universidade de São Paulo. Ribeirão Preto, São Paulo, Brasil; V Universidade Federal do Piauí. Teresina, Piauí, Brasil

**Keywords:** Pessoas em Situação de Rua, Saúde da Mulher, Cuidado Pré-Natal, Acesso aos Serviços de Saúde, Enfermeiras e Enfermeiros

## Abstract

**Objetivos:**

compreender a perspectiva de enfermeiros acerca da acessibilidade de gestantes em situação de rua ao cuidado pré-natal.

**Métodos:**

estudo qualitativo, com análise baseada no conceito de acessibilidade. Foram realizadas entrevistas semiestruturadas, com 11 enfermeiros que trabalham no Consultório na Rua na região Norte do Brasil.

**Resultados:**

enfermeiros se deparam com barreiras geográficas e situações de perigo nas regiões de fronteira, reconhecendo que há um contexto de violência física, sexual, psicológica que envolve gestantes em situação de rua que procuram atendimento no Consultório na Rua. A atuação dos enfermeiros do Consultório na Rua ocorre articulada a outros serviços da Rede de Atenção à Saúde. A implementação de medidas educativas é uma estratégia potente, assim como o estabelecimento de vínculos com mulheres.

**Considerações Finais:**

a atuação do Consultório na Rua proporciona encontro com as gestantes *in loco* no território, o que pode propiciar a acessibilidade geográfica e socio-organizacional ao cuidado pré-natal.

## INTRODUÇÃO

O acesso à assistência pré-natal é um direito da mulher no Brasil, e a acessibilidade a cuidados maternos de qualidade no pré-natal pode impedir a ocorrência de mortes maternas evitáveis^(1)^. A Organização das Nações Unidas (ONU) e a Organização Mundial da Saúde (OMS) têm buscado diminuir esse indicador definindo compromissos globais, como os Objetivos de Desenvolvimento Sustentável (ODS)^(2)^. A meta 3.1 dos ODS referente à redução da razão de mortalidade materna (RMM) estabeleceu reduzir a RMM global para menos de 70 mortes maternas por 100.000 nascidos vivos até 2030^(2)^.

Uma das estratégias apontadas para a redução da RMM é o cuidado pré-natal^(1)^. No entanto, o acesso aos serviços de saúde e ao cuidado pré-natal é limitado para muitas gestantes, sobretudo àquelas que se encontram em situação de vulnerabilidade, como as pertencentes a minorias étnicas, em situação de pobreza, em situação de rua, migrantes e refugiadas, profissionais do sexo, mulheres expostas à violência doméstica, portadoras de agravos de saúde mental, usuárias de substâncias, entre outras^(3-4)^. Tais iniquidades de acessibilidade aos serviços de saúde entre as mulheres refletem o perfil da mortalidade materna, com predominância de mulheres negras, de baixo nível socioeconômico e com baixa escolaridade^(3)^.

Definem-se pessoas em situação de rua como aquelas que vivem em condições de extrema pobreza, que romperam o vínculo familiar, que não possuem moradia convencional e ocupam espaços públicos para habitar e buscar formas de sustento, de forma temporária ou permanente, além de utilizar locais de acolhimento para pernoite^(5)^. As mulheres representam aproximadamente 18% da população em situação de rua no Brasil, e parcela significativa dessas concentra-se na faixa etária de 18 a 55 anos, muitas delas no período reprodutivo^(5)^. A precariedade das condições sociais e de saúde favorece gestações não intencionais, demonstrando falhas nas ações de planejamento reprodutivo, além de contribuir para abortos inseguros, aumentando o risco de morbidade e mortalidade materna^(6-8)^.

No contexto brasileiro, evidencia-se a configuração intergeracional de famílias em situação de rua^(9)^, contribuindo para a perpetuação do ciclo da pobreza e da miséria. Ademais, barreiras como preconceitos e estigmas produzidos, falta de recursos financeiros, falta de mobilidade e inexistência de programas direcionados às gestantes em situação de rua evidenciam a dificuldade de acessibilidade de mulheres para a realização do pré-natal^(9)^, representando fatores de risco para a saúde do binômio gestante-feto^(4,7-8,10)^. Devem-se considerar o efeito do álcool, do tabaco, o uso de substâncias ilícitas, a baixa ingesta nutricional, as comorbidades maternas, a exposição à poluição e os fatores ambientais, além de violência nas ruas e práticas sexuais inseguras como fatores agravantes para a saúde do binômio gestante-feto^(4,7,10-12)^.

Diante de tais desafios, a equipe de Consultório na Rua (CnaR) busca articular e prestar assistência integral à saúde da população em situação de rua no território, com a incorporação de práticas de prevenção de doenças e promoção da saúde e ampliação do acesso às redes de atenção, além de assegurar os direitos dessa população^(13)^. O CnaR atua de forma itinerante, desenvolvendo ações *in loco* no território vivo das ruas, construindo seu *setting* terapêutico fundamentado no vínculo e no acolhimento da população em situação de rua segundo as especificidades, o contexto e as características do território^(13-14)^. O CnaR está em consonância com o ODS 3, que busca assegurar uma vida saudável e promover o bem-estar para todos, sem deixar ninguém para trás^(2)^.

O trabalho da enfermagem reflete o cuidado aos indivíduos, às famílias e à comunidade, com o desempenho de atividades assistenciais, gerenciais, educativas e investigativas para promoção, manutenção e recuperação da saúde individual e coletiva^(15)^. Nessa direção, destaca-se a atuação dos enfermeiros, enquanto integrantes das equipes de CnaR, para contribuir com a acessibilidade de gestantes em situação de rua ao cuidado pré-natal, tendo em vista as suas vulnerabilidades e necessidades de cuidado diferenciadas em relação à população em geral.

Nesse contexto, torna-se imprescindível estudar o processo de trabalho do enfermeiro no cuidado pré-natal no âmbito do CnaR para maior compreensão do seu papel no cuidado às mulheres em situação de rua no período gravídico-puerperal. A realização de tais estudos tem o potencial de identificar a compreensão do enfermeiro sobre o objeto, os instrumentos utilizados, a finalidade e o produto final obtido com as ações desenvolvidas por esse profissional^(15)^. Diante do exposto, o presente estudo busca fomentar reflexões considerando o cotidiano do trabalho do enfermeiro junto a gestantes em situação de rua no CnaR, contribuindo para o alcance dos ODS e direcionamento de políticas públicas para essa população.

## OBJETIVOS

Compreender a perspectiva de enfermeiros acerca da acessibilidade de gestantes em situação de rua ao cuidado pré-natal.

## MÉTODOS

### Aspectos éticos

O estudo seguiu os preceitos éticos especificados na Resolução nº 466, de 12 de dezembro de 2012, do Conselho Nacional de Saúde para pesquisas realizadas com seres humanos. A pesquisa foi aprovada por um Comitê de Ética em Pesquisa vinculado à Comissão Nacional de Ética em Pesquisa (CONEP) em maio de 2022.

### Tipo e cenário de estudo

Trata-se de pesquisa com abordagem qualitativa^(16)^. O escopo da pesquisa foi orientado pelas diretrizes do *COnsolidated criteria for REporting Qualitative research* (COREQ)^(17)^. O estudo fundamentou-se nas bases conceituais da acessibilidade propostas por Donabedian^(18)^. Entende-se a acessibilidade como a capacidade de produzir ofertas e de responder às necessidades da população, considerando as características dos serviços e dos recursos de saúde que contribuem ou representam obstáculos para a utilização pelos usuários^(18)^. Esse autor distingue duas dimensões da acessibilidade, sendo que a acessibilidade socio-organizacional refere-se às características dos recursos ofertados e a acessibilidade geográfica envolve as características relacionadas à distância e ao tempo necessárias para alcançar e obter os serviços^(18)^.

O cenário do estudo foram os serviços de CnaR nos estados do Acre, Amapá, Amazonas, Pará e Tocantins, situados na região Norte do Brasil. Tais serviços atendem à população em situação de rua, profissionais do sexo, migrantes, indígenas *Warao* e outras populações em situação de vulnerabilidade no território. De acordo com a quantidade e composição profissional, as equipes de CnaR são classificadas em modalidades I, II e III. Na modalidade I, a equipe é composta por quatro profissionais, dois de nível superior e dois de nível técnico. Na modalidade II, são seis profissionais, três de nível superior e três de nível técnico. A modalidade III apresenta a mesma constituição que a II, acrescida do profissional médico^(19)^. No presente estudo, foram incluídas equipes de CnaR das três modalidades, compostas pelos profissionais, tais como enfermeiros, médicos, psicólogos, assistentes sociais, nutricionistas, educadores físicos, técnicos de enfermagem, técnicos de saúde bucal e agentes sociais.

### População e critérios de seleção

A população de referência foi constituída por enfermeiros que trabalhavam em equipes do CnaR na região Norte do Brasil. Os critérios de inclusão foram enfermeiros que estivessem atuando no CnaR por pelo menos seis meses e que realizassem cuidados pré-natais. A amostra foi obtida pelo processo de amostragem por conveniência. Inicialmente, foi solicitado às secretarias estaduais e municipais da saúde o contato dos coordenadores dos CnaR, para que, via e-mail, a pesquisa lhes fosse apresentada e fosse acertada a intermediação do acesso dos pesquisadores aos enfermeiros do CnaR, potenciais participantes desta pesquisa. Subsequentemente, foram enviados e-mails para todos os enfermeiros que atendessem aos critérios de inclusão. O período de coleta de dados ocorreu entre outubro de 2022 e janeiro de 2024.

### Coleta de dados

Foi realizado estudo piloto com o objetivo de identificar dificuldades, aperfeiçoar a aplicação do instrumento, e não houve necessidade de modificações. As entrevistas de sensibilização foram conduzidas por uma das autoras, enfermeira, com titulação de doutorado e experiência em pesquisa qualitativa.

Os dados foram obtidos entre outubro de 2022 e janeiro de 2024 por meio de entrevista semiestruturada, realizada remotamente e gravada na plataforma de videoconferência *Google Meet* ^®^. Após a manifestação de interesse e concordância em participar do estudo, mediante assinatura do Termo de Consentimento Livre e Esclarecido, uma das pesquisadoras entrou contato com o enfermeiro para o agendamento da entrevista. As entrevistas foram realizadas por duas pesquisadoras enfermeiras, com títulos de doutorado e mestrado, com experiência em pesquisa qualitativa, e uma acadêmica de enfermagem capacitada para a função. As entrevistas tiveram duração média de 40 minutos e foram realizadas uma única vez por participante.

As entrevistadoras não tinham contato prévio com os serviços e/ou com os participantes. O número de participantes foi definido por meio do critério de saturação dos dados, com a inclusão de 11 enfermeiros. O critério de saturação dos dados envolveu a percepção do pesquisador durante o processo contínuo de análise dos dados. Diante das repetições das falas e da ausência de novos pronunciamentos, procedeu-se à finalização das entrevistas^(20)^.

O instrumento de coleta de dados seguiu roteiro semiestruturado composto por duas partes: a primeira composta por questões fechadas, referentes à caracterização sociodemográfica das participantes, incluindo idade, cor autorreferida, nível de formação (especialização, mestrado, doutorado), tempo de atuação no CnaR; e a segunda composta por questões abertas relacionadas ao cuidado do enfermeiro às mulheres em situação de rua. Foram realizadas as perguntas norteadoras: como é a abordagem à gestante no CnaR? Como ocorre o cuidado do enfermeiro no pré-natal às mulheres em situação de rua?

Após a coleta dos dados, procedeu-se à transcrição das entrevistas. Para garantia do sigilo e confidencialidade dos participantes, as entrevistas foram identificadas a partir da letra “E”, de enfermeiro, seguida de número ordinal, de acordo com a ordem de realização das entrevistas.

### Análise dos dados

Os dados foram apresentados a partir da análise temática indutiva^(21)^, a partir de seis etapas, que são: 1) familiarização com os dados; 2) geração de códigos iniciais; 3) busca dos temas; 4) revisão dos temas; 5) definição e nomeação dos temas; 6) produção de relatório e analisados à luz do conceito de acessibilidade (Quadro 1).

**Quadro 1:** Codificação dos dados da pesquisa, Macapá, Amapá, Brasil, 2024

Códigos iniciais	Códigos intermediários	Códigos final
Atuação *in loco*	Contexto geográfico e segurança	Desafios da acessibilidade de gestantes em situação de rua ao cuidado pré-natal
Barreiras na acessibilidade geográfica		
Questões de segurança e periculosidade do território		
Migração das mulheres no território		
Particularidades das regiões de fronteira	Migração e refugiados em situação de vulnerabilidade social	
Movimentos migratórios de imigrantes e refugiados		
Contexto de vulnerabilidade, captação tardia da gestante	Vulnerabilidade social e saúde	A influência do contexto de vida da gestante em situação de rua na acessibilidade ao cuidado pré-natal
Dependência de álcool e outras drogas		
Alimentação escassa		
Prostituição, sexo transacional		
Violência física, sexual, psíquica, simbólica e estatais	Violência e abuso	
Descontinuidade da gravidez, desfechos obstétricos desfavoráveis	Saúde sexual e reprodutiva	
Limitada disposição de recursos sociais	Acesso aos recursos sociais e serviços de saúde	
Desafios na integração da Rede de Atenção à Saúde		
Oferta de maior número de consultas em intervalos menores	Acessibilidade e qualidade do cuidado do pré-natal	As potencialidades da atuação do enfermeiro no cuidado pré-natal para a acessibilidade das gestantes em situação de rua
Acessibilidade ao cuidado pré-natal		
Conscientização dos direitos das gestantes	Promoção da saúde materna e neonatal	
Abordagem de riscos		
Redução de danos		
Criação de vínculos	Desenvolvimento de relacionamentos terapêuticos	

## RESULTADOS

Dos 11 enfermeiros participantes, oito eram mulheres e três eram homens, com média de idade de 35 anos. Além disso, seis se identificavam como pardos e seis eram casados/em relacionamento estável. O tempo médio de formação foi de nove anos; nove possuíam especialização; e dois possuíam mestrado. O tempo médio de atuação no CnaR foi de seis anos, com vínculo por meio de contratos ou concurso público. A maioria dos serviços de CnaR possuía unidades móveis para o atendimento, como vans, e apenas um serviço possuía carro de transporte da equipe.

### Desafios da acessibilidade de gestantes em situação de rua ao cuidado pré-natal

A dimensão organizacional do trabalho dos enfermeiros da equipe do CnaR tem o potencial de alcançar as gestantes que vivenciam a situação de rua por meio da atuação *in loco,* para promover a acessibilidade ao cuidado pré-natal. Para tanto, o enfermeiro tem como estratégia a utilização dos equipamentos sociais do território para o cuidado a essas mulheres, destacando as Unidades Básicas de Saúde (UBS), articulando-as à Rede de Atenção à Saúde.

*A gente vai na rota e tenta trazer, porque a dificuldade delas é ir até a UBS. Porque, às vezes, fica longe, essas coisas assim, e a gente já tenta trazer ela dentro do carro do consultório na rua até a UBS pra fazer esse atendimento, ir lá, né? Muitas vezes, tem uma equipe de Estratégia da Família que atende lá próximo do local que elas ficam*. (E4)

*A gente trabalha muito em equipe, mas eu faço pré-natal na rua. Como não temos van* [unidade móvel], *quando eu sinto a necessidade de fazer uma avaliação, por exemplo, palpação, altura uterina, vamos na Unidade de Saúde da Família mais próxima do território que ela tá inserida para fazer uma avaliação específica, testagem rápida, vacinação.* (E5)

No entanto, existem barreiras na acessibilidade geográfica devido aos deslocamentos da mulher para outros territórios. Ressalta-se também a periculosidade de determinadas regiões, o que impede a equipe do CnaR adentrar por questões de segurança, dificultando o encontro dos profissionais com as gestantes para o cuidado pré-natal.

*Nós fazemos os exames iniciais e já encaminhamos para a Unidade Básica de Saúde, e ficamos acompanhando* [...] *só que elas acabam sumindo. No decorrer desses nove meses, elas mudam de território, e aí, quando a gente vai ver, já terminou o período de pré-natal e ela já aparece com o filho no colo.* (E3)

*Elas não têm uma adesão ao pré-natal. Eu estou com uma paciente que eu estou há dois meses querendo trazer ela pra fazer o acompanhamento do pré-natal. E toda vez que eu vou na rota, eu não consigo encontrar ela, e aí fica difícil pra gente fazer esse acompanhamento. Já era pra estar tomando o sulfato ferroso e ácido fólico.* (E4)

*Dependendo dos locais que elas estão, tem alguns que a gente não adentra, por segurança da equipe. Então, a gente lida mesmo com o interesse, com a disponibilização delas mesmo de querer. É um trabalho de conseguir conscientizar elas da importância de realizarem o pré-natal.* (E1)

Destaca-se ainda que as regiões de fronteira foram postas como áreas desafiadoras ao cuidado, que recebem os impactos dos fenômenos sociais relacionados aos movimentos migratórios de imigrantes e refugiados, com destaque para indígenas da etnia *Warao* provenientes da Venezuela, de forma intensificada.

*É complicado principalmente para quem está na área de fronteira, porque acaba recebendo e lidando com muita informação. A grande maioria delas são indígenas, então, quando elas vêm de lá da Venezuela, elas vêm fugindo da questão* [da crise econômica] *do país. O município está imerso em grande crise, porque elas vêm da floresta. Elas são indígenas, elas vão fazer o que chamam na rua de “manguear”, que é pedir. Elas estão na rua, outras conseguem ir até um local, pra ficar no abrigo, mas passa o dia todo vivendo como a população* [em situação] *de rua aqui.* (E1)

*Quem sofre os impactos* [da imigração] *de primeira é quem está na fronteira. A maioria dos imigrantes na rua vem da Venezuela, são indígenas, e o registro é bem complicado, e também de se entrar no território.* (E3)

A assistência pré-natal realizada pelo enfermeiro às mulheres em situação de rua, muitas vezes, não acompanha o calendário recomendado pelo Ministério da Saúde, devido à identificação tardia da gravidez pelas mulheres. A captação tardia da gestante, quando no terceiro trimestre, direciona os primeiros atendimentos para os pontos de atenção secundária e terciária, limitando-se às tecnologias duras que podem detectar maiores ou menores riscos.

*Eu agendava os pré-natais delas na UBS, fazia a busca ativa, nenhuma foi captação precoce, já foi com 20 semanas em diante.* (E10)

*Das mulheres, quando já estão gestantes, a gente tenta fazer o pré-natal delas, mas geralmente a gente já fica sabendo da gestação quando elas já estão quase pra ter* [o parto]. *Então, assim, geralmente, o pré-natal, ele não existe; o pré-natal, ele já é diretamente pra ir pra maternidade.* (E1)

[...] *tem algumas que a gente só vai saber da gravidez no final da gestação. Então, a gente já tenta levar ela para o hospital* [acompanhamento de alto risco]*. No dia que a gente encontra, faz ultrassom, vê qual a semana que está, faz os testes rápidos para saber se tem alguma doença, a gente faz o que a gente pode.* (E2)

### A influência do contexto de vida da gestante em situação de rua na acessibilidade ao cuidado pré-natal

O reconhecimento, por parte dos profissionais, que as mulheres em situação de rua estão inseridas em um contexto de violência física, sexual, psíquica, simbólicas e estatais, favorecendo a dependência de álcool, tabaco e outras drogas ilícitas, com alimentação escassa, prostituição, prática do sexo para subsistência, entre outras especificidades, é fundamental para articular o cuidado de enfermagem e a acessibilidade socio-organizacional.

*As mulheres já são muito expostas, mas essas mulheres que vivem na rua, elas ficam mais ainda, porque elas sofrem violência, muitas delas sofrem violência sexual.* [...] *elas ficam na praça ou algum ponto comercial sozinhas, e elas acabam fazendo uso de álcool e outras drogas ainda estando grávida. As mulheres que eu atendo, a maioria delas são usuárias de álcool, e aí a gente tenta orientar elas nesse período.* (E4)

*Muitas mulheres trazem o desejo de gestar, uma necessidade de saúde que eu identifico enquanto enfermeira. Mulheres que sofrem múltiplas violências na rua, tanto relacionada à sexual, física e moral por parte inclusive de agentes públicos.* (E5)

*A gente tem uma grávida de gêmeas, e aí a gente tá acompanhando esse pré-natal. Ela tá ainda em situação de rua, ela tá dormindo na rua mesmo, e aí ela continua fazendo uso de droga, continua se prostituindo.* (E6)

*A maioria são usuárias de drogas e, mesmo na gestação, elas continuam usando drogas e bebendo.* (E10)

A interrupção da gravidez foi mencionada pelos enfermeiros como frequente e as causas como sendo de difícil identificação. O contexto de vida nas ruas pode favorecer o desfecho do abortamento, seja pelas precárias condições de saúde das mulheres ou pela violência a que estão expostas nas ruas.

*Em relação ao abortamento, a gente tem muitos relatos das mulheres* [...] *o complicado é que a gente não sabe de fato* [como ocorreu], *como elas só informam depois, a gente não sabe se foi uma síndrome alcoólica fetal, não sabe dizer se foi algum maus-tratos, se foi violência, o que de fato foi, tentar identificar essa causa. Na maioria, a gente tem os relatos da própria mulher.* (E7)

*A paciente me relatou que ela se envolveu numa briga com outra mulher, e aí ela levou chute na barriga. Ela já estava com oito meses e perdeu o bebê.* (E6)

*Aí fizemos* [o teste de gravidez], *deu positivo. Ela na questão de não aceitação: “Eu não aceito porque não posso ter”. Aquela coisa toda e creio que ficou tentando abortar, não sei de que maneira, chegava na semana seguinte queixando de dores abdominais e queixando de sangramento e a gente enquanto equipe conduzia para o hospital de referência.* (E3)

Ainda, destacam-se a limitada disposição de recursos sociais para as gestantes em situação de rua e a falta de integração da Rede de Atenção à Saúde para o cuidado humanizado da mulher em situação de rua no ciclo gravídico-puerperal.

*Um dos nossos desafios com as mulheres em situação de rua é em relação à oferta de cuidados gerais, mas principalmente a sexualidade, reprodutividade. Elas têm o desejo de gestar e, infelizmente, quando a gente fala em rede* [no município], *nós não temos nenhum suporte para acolhimento de população em situação de rua. Nós não temos albergue, nós não temos centro pop, nós não temos casa de passagem. E é onde a gente entra na perspectiva da redução de danos. De dizer pra ela que o fato dela tá inserida num contexto vulnerável, ela já se coloca em risco, já está ainda mais, né?* (E5)

*Eu fiquei chateada quando eu cheguei na maternidade, porque ela pariu sozinha. Não deixaram o companheiro dela entrar. Não existe isso, só porque é uma pessoa em situação de rua, a pessoa tem que parir sozinha?* (E10)

### As potencialidades da atuação do enfermeiro no cuidado pré-natal para a acessibilidade das gestantes em situação de rua

Na atenção pré-natal, destaca-se o papel do enfermeiro na conscientização das gestantes dos seus direitos de ter acesso aos serviços de saúde e de serem acolhidas e atendidas com dignidade e respeito.

*É a questão da garantia dos direitos. Elas entenderem que elas têm direitos e elas possam se empoderar disso. A gente tenta muito empoderar elas: que elas têm direito a fazer um pré-natal na unidade de saúde, não é porque eu estou fazendo pré-natal dela na rua que ela não tem direito de ir na unidade de saúde.* (E7)

Diante das complexas necessidades de saúde das gestantes em situação de rua, a atuação do CnaR para a acessibilidade ao cuidado pré-natal ocorre em conjunto e articulação com os diferentes serviços que integram a Rede de Atenção à Saúde, com o desenvolvimento de ações integradas e compartilhadas com as UBS, as Estratégias Saúde da Família (ESF), os Centros de Atenção Psicossocial (CAPS), os serviços de urgência e emergência e de outros níveis de atenção, de acordo com a necessidade da mulher. Uma estratégia utilizada pelos enfermeiros foi oportunizar um maior número de consultas, em intervalos menores, para o acompanhamento da gestante.

*O pré-natal, ele acontece de uma forma muito clara, segue padrões, mesmo sendo no consultório na rua. A gente faz todo e qualquer exame, a gente marca ultrassom, tem as vitaminas que essa mulher tem que tomar ao longo desses nove meses e, se for num pré-natal normal, vamos assim dizer,* [fará o acompanhamento] *na Estratégia Saúde da Família. Essa mulher vai uma vez com o médico, uma vez com a enfermeira.* [...] *por ser uma mulher vulnerável, eu faço esse retorno em um curto espaço de tempo, aí eu consigo ter um acompanhamento melhor, mais de perto com ela. Ela sempre vai estar ali, aos meus olhos.* [...] *a gente leva pro hospital de referência, garante esse atendimento.* (E6)

[...] *aqui, a gente tem esse cuidado pra que ela não fique em situação de rua durante toda a gravidez: ela hospitaliza no CAPS AD* [Centro de Atenção Psicossocial Álcool e Drogas] *se ela for usuária de álcool e drogas. E as duas* [gestantes] *que nós tivemos, infelizmente elas eram dependentes.* (E8)

*Quando elas seguem até o final com a gente, elas recebem o acompanhamento até o hospital, até uma maternidade para parto de alto risco. Elas acabam sendo classificadas como alto risco, por estar em situação de rua e por não manter um pré-natal direitinho, ficar bem alternado: as consultas, os exames, vitaminas, vacinas, fica uma coisa bem bagunçada, e aí a gente consegue encaminhar e acompanhar no pós-parto e dar o seguimento que é necessário para elas.* (E3)

A implementação de medidas educativas é uma estratégia potente para o cuidado do enfermeiro e promover a acessibilidade socio-organizacional durante o cuidado pré-natal das gestantes em situação de rua. A abordagem dos riscos de determinadas práticas nocivas no período gestacional tem o potencial de contribuir para a conscientização e mudança de hábitos, na perspectiva de redução de danos à saúde do binômio.

*Eu converso muito com elas sobre os problemas que ela pode adquirir tanto na gravidez como quando a criança nascer, por ela estar fazendo esse uso dessas substâncias. A gente conversa bastante com elas.* (E4)

*É uma conquista gradual tanto pra ela* [usuária acompanhada no CnaR], *que está ganhando consciência da gestação dela. Ela relata que ela está utilizando drogas em menor quantidade. Então, é uma conquista pro Consultório na Rua saber que a gente está transformando pequenas realidades que vai gerar um impacto grande pra vida dela. Pra mim, é uma das melhores coisas saber que a gente está podendo transformar, porque o conhecimento é transformador.* (E7)

Denota-se a criação de vínculos entre o enfermeiro e a mulher como elemento fundamental do cuidado, com repercussões na sua adesão ao cuidado pré-natal.

*Inclusive, eu estou com uma paciente, uma paciente que é assim, difícil, porque ela é tão difícil, que a gente gosta dela. Ela é apaixonante de tão difícil. Ela está no segundo filho dela. No primeiro, o pré-natal ela fez todo no Consultório na Rua. É uma paciente que eu particularmente me emociono porque ela gerou um vínculo muito grande com a equipe.* [...] *ela está na quinta consulta dela, isso ela está com 28 semanas, a gente vai conseguir fazer mais de seis consultas. A gente conseguiu fazer teste rápido, consegue “suplementar ela”, a gente tá conseguindo dar bastante orientação.* (E7)

Nessa perspectiva, a atuação do enfermeiro no cuidado pré-natal de gestantes em situação de rua tem o potencial de promover o acesso ao sistema de saúde e prover resultados satisfatórios dentro das condições possíveis das mulheres.

*Ela chegou com um baixo peso muito característico, ela está ganhando um peso. Então, a gente está vendo o quanto ela está melhorando, o quanto ela se sente melhor, o quanto ela procura a consulta pré-natal, ela fala com a gente na rua: “Falta uns dias pra minha consulta!”. A gente saber que a pessoa quer receber uma consulta é a coisa mais gratificante que tem, porque normalmente a gente fica nessa captação, nessa busca ativa. O fato de ela procurar o atendimento e ela estar meio que dentro do padrão de normalidade, dentro do mês, dentro dia da consulta, conforme preconizado mensalmente, a cada 15 dias, uma vez por semana e ela conseguir cumprir isso, é uma coisa muito boa.* (E7)

## DISCUSSÃO

As condições das gestantes em situação de rua infringem os direitos humanos, sexuais e reprodutivos e de uma vida com dignidade. A gestação em situação de rua refere-se a uma condição de risco e vulnerabilidade social, contexto de precariedade da vida, ruptura de vínculos afetivos e sociais, e configura-se um espaço inadequado para gestar^(8)^. Deve-se ressaltar a exposição das mulheres às situações de violência nas ruas, abuso sexual, exposição às infecções sexualmente transmissíveis (IST), uso abusivo de substâncias, rejeição das famílias mediante a gravidez, vergonha, tristeza, insatisfação, culpa pela gestação não intencional e incertezas em relação ao futuro^(7-9,13,22)^.

O presente estudo identificou que a atuação dos enfermeiros *in loco* no CnaR contribuiu para a acessibilidade socio-organizacional e geográfica das gestantes em situação de rua, constituindo, muitas vezes, a porta de entrada aos serviços de saúde para o cuidado pré-natal. As ações na rua e a busca ativa representam dispositivos essenciais para a garantia de acesso ao cuidado^(14)^.

A prática em trânsito mobiliza o encontro entre diferentes trabalhadores e percorre as redes de saúde e intersetorial, buscando a articulação para o atendimento de pessoas que encontravam-se invisíveis nos/aos cenários do Sistema Único de Saúde^(14,23)^.

Ao considerar os riscos de uma gestação em situação de rua, destaca-se a necessidade de integração entre os serviços de Atenção Primária à Saúde, bem como cuidados de equipe de saúde especializada e multiprofissional, em serviços de referência secundário ou terciário^(22,24)^. No entanto, a articulação entre o CnaR e os serviços que compõem a Rede de Atenção à Saúde e a rede intersetorial podem se dar de forma fragilizada, considerando as dificuldades dos serviços em acolher as demandas mais complexas das pessoas em situação de rua e a ausência de fluxos formalizados entre os equipamentos do território^(14)^.

O início tardio, as dificuldades no seguimento, ou a inexistência do cuidado pré-natal foram realidades relatadas pelos enfermeiros, dado que a acessibilidade, como a oferta do cuidado, pode auxiliar no entendimento das variações do uso do serviço de saúde por grupos populacionais^(18)^. Identificou-se a dificuldade da gestante em situação de rua em ter o primeiro contato com o profissional, iniciar e/ou dar continuidade ao pré-natal, além da falta de conhecimento das mulheres sobre onde buscar os serviços^(4)^. Adicionalmente, a baixa vinculação da população em situação de rua com os serviços de Atenção Primária à Saúde faz com que a porta de entrada aos serviços de saúde se dê predominantemente por meio de urgência e emergência, com lacunas na continuidade do cuidado^(14,25)^.

As barreiras para a acessibilidade das gestantes em situação de rua aos serviços de saúde podem relacionar-se à estrutura e inadequação do sistema de saúde às necessidades e especificidades desse grupo^(7,14)^, considerando também a adequação dos profissionais e dos recursos utilizados para a assistência desse grupo^(18)^. Além disso, a acessibilidade, considerando a localização dos serviços e falta de transporte, faz parte da realidade das pessoas que vivem nas ruas^(7)^. Usualmente, as gestantes nessa situação necessitam de auxílios de outras pessoas para conseguir transporte ou precisam caminhar longos percursos para as consultas de pré-natal, o que resulta no absenteísmo^(7)^. Os relatos dos enfermeiros mostraram que o CnaR pode utilizar dispositivos próprios para o transporte das gestantes em situação de rua, quando disponíveis, bem como os dispositivos da Rede de Atenção à Saúde e de equipamentos sociais do território, com destaque às UBS, para promover a acessibilidade ao cuidado pré-natal a essas gestantes.

Destaca-se, ainda, que as regiões de fronteiras foram descritas como cenários fragilizados pelo crescente movimento migratório para o Brasil, com a necessidade de adequar-se à demanda imediata que lhes é imputada, muitas vezes, decorrendo na sobrecarga dos sistemas de saúde^(26)^. A Venezuela vivencia uma crise política, econômica e social, e 33 venezuelanos, em média, entram no Brasil por hora, aproximadamente, 800 por dia. Entre as mulheres imigrantes venezuelanas, um grupo muito expressivo que migrou para a região norte do Brasil, sobretudo para as capitais de Manaus, Belém e Boa Vista, são as mulheres indígenas da etnia *Warao* ^(27)^.

Gestantes imigrantes são menos propensas a realizar o cuidado pré-natal de forma adequada, e há risco aumentado de obter desfechos adversos no período gravídico puerperal^(28)^. Nesse contexto, a competência cultural dos profissionais de saúde desempenha um papel fundamental na garantia de cuidados de saúde eficazes e culturalmente sensíveis. No entanto, é importante reconhecer que a competência cultural vai além do conhecimento superficial das tradições e costumes dos refugiados, pois envolve uma compreensão profunda das suas experiências, necessidades e perspectivas únicas, bem como o reconhecimento e respeito pela sua diversidade cultural. Além disso, é fundamental considerar o impacto do trauma e das experiências prévias de cuidados de saúde nos refugiados, que podem influenciar suas atitudes e comportamentos em relação aos serviços de saúde^(26)^. Torna-se extremamente urgente e importante a realização de treinamentos para familiarizar os profissionais de saúde com a língua e com os costumes das imigrantes, principalmente venezuelanas, a fim de promover um cuidado transcultural que abarque a diversidade dos indivíduos^(29)^, em vista de conquistar a acessibilidade e universalidade dos serviços e respeitar as especificidades da medicina tradicional praticada por esses povos.

Os tensionamentos e a falta de confiança das usuárias nos profissionais de saúde, já que essas, muitas vezes, apresentam problemas na justiça^(14)^, além do tratamento de maneira inferiorizada, do preconceito, do estigma, da exclusão, das barreiras de linguagem e de cultura e medo, podem corroborar a insatisfação, a fragilidade do cuidado, o retardo na busca e a continuidade do cuidado pré-natal pelas gestantes em situação de rua, agravando as condições de vulnerabilidade^(4,7-8)^. Ainda, experiências negativas pregressas e o receio de perder a custódia da criança podem influenciar as mulheres a não buscarem os serviços de saúde durante a gestação^(7)^. Assim, deve-se considerar que a atenção à saúde de gestantes em situação de rua não remete apenas a fatores individuais, mas a todo o sistema político-socioeconômico no qual estão inseridos os serviços de saúde^(7,25)^.

Devido à complexidade que envolve a gestação em situação de rua, torna-se imprescindível considerar que as necessidades dessa população são diferentes e, consequentemente, requerem recursos/métodos também diferenciados para o seu atendimento^(25)^. Nesse sentido, as usuárias podem precisar de tecnologias de cuidado nem sempre habituais nas rotinas dos profissionais de saúde, que perpassam as condutas protocolizadas que tendem a uniformizar as ofertas de cuidado^(23)^. Ressalta-se que gestantes em situação de rua vivenciam a fragilidade no cuidado centrado na pessoa e a dificuldade da acessibilidade geográfica aos serviços de saúde, além da falta de atendimento individualizado, considerando que as necessidades e singularidades dessa população permanecem um desafio para os sistemas de saúde^(7)^. A forma de organização dos serviços para responder aos desafios colocados pelas mulheres tem o potencial de impactar melhores desfechos gestacionais para além dos limites das práticas biomédicas e assistenciais, com a garantia de direitos das mulheres e de seus filhos^(30)^.

Fez-se necessária a oferta de meios legais de assistência pelo poder público, com espaços e ações que possam reduzir os estigmas, a dor, o sofrimento e a exclusão social^(8)^. Ainda, foi necessária a elaboração de estratégias de enfrentamento da condição de gestar nas ruas, bem como de fomentar cuidados que valorizem os modos de existir dessas mulheres, com a implementação de práticas e procedimentos em saúde que propiciem a promoção da saúde, a prevenção de doenças, o diagnóstico precoce^(8)^, e o fortalecimento da rede social e suporte às gestantes^(7)^.

No presente estudo, foi identificado que o baixo nível de conhecimento e letramento em saúde são fatores prejudiciais para o acompanhamento pré-natal e pós-natal de mulheres em situação de rua. Destacam-se importantes limitações quanto aos recursos para obtenção de informações, sendo que muitas mulheres desconhecem os riscos do uso de álcool e substâncias para o crescimento e desenvolvimento do feto^(7)^. Nessa perspectiva, as estratégias de educação em saúde realizadas pelos enfermeiros do CnaR contribuem para o acesso dessas gestantes a informações sobre as condições de saúde, riscos e a prevenção de agravos.

Concomitante às orientações sobre os cuidados durante a gestação, tornam-se fundamentais a vinculação ao local do parto, a garantia de acesso qualificado a esse local e a um parto humanizado, o cuidado à mulher no período pós-parto e ao recém-nascido, com a oferta de planejamento reprodutivo pós-parto, além de articulações intersetoriais necessárias de acordo com as demandas, como acompanhamento por serviços socioassistenciais, recebimento de benefícios ou transferência de renda, inserção em programas habitacionais, entre outros^(31)^.

As condições de vulnerabilidade concernentes às mulheres em situação de rua expressam-se em múltiplas necessidades de saúde^(11)^. Vivenciar a maternidade nesse contexto é extremamente complexo, marcada por profundas relações desiguais de classe, raça e gênero^(7,30)^. Prevalece um contexto de dubiedade entre a defesa dos direitos das mulheres e a defesa dos fetos/crianças. A criminalização e julgamentos em relação à inaptidão das mulheres em situação de rua para o exercício da maternidade representam uma tendência em que a proteção do feto/criança implica a retração dos direitos das mulheres. Durante o pré-natal, o uso de dispositivos jurídicos e normativos centrados na punição gera desconfiança, propiciando o abandono e a recusa das mulheres na busca dos serviços de assistência social e de saúde^(30)^. Embora a condição das ruas se distancie do ideal para o exercício da maternidade, devem-se considerar o desejo da mulher de maternar e a necessidade de políticas públicas que amparem as mulheres, famílias e crianças ancoradas na perspectiva dos direitos humanos^(30)^.

Considera-se que a solução potencial para reduzir os índices de gestações não intencionais nas mulheres em situação de rua seria o acesso a ações de planejamento reprodutivo, que inclui o aconselhamento. Essa é uma ação do enfermeiro, em que são ofertadas informações em linguagem compreensível, sobretudo em relação aos métodos efetivos de contracepção, incluindo os reversíveis de longa duração, e as formas para o acesso aos métodos contraceptivos, caso seja o desejo da mulher^(32)^. As recomendações de aconselhamento e planejamento reprodutivo podem ser oportunizadas durante o pré-natal e, ainda, no pós-parto imediato^(33)^.

Para a garantia da acessibilidade aos serviços de saúde, fazem-se necessários a capacitação dos profissionais para acolher as mulheres em situação de rua^(8)^, o conhecimento acerca da Política Nacional para a População em Situação de Rua e a sensibilização acerca dos direitos dessas usuárias, uma vez que o acesso universal está assegurado na Constituição Federal^(13-14,25)^. É notório, diante dos resultados deste estudo, que os ODS 1- Erradicação da pobreza, ODS 3 - Saúde e bem-estar e ODS 5- Igualdade de gênero ainda necessitem de esforço de políticas públicas para que sejam atingidos no Brasil.

O cuidado pré-natal propiciado pelo CnaR pode auxiliar no alcance de alguns ODS. O ODS 1 menciona acabar com a pobreza em todas as suas formas, em todos os lugares. Sobre esse aspecto, sabe-se que a relação entre pobreza e saúde está bem estabelecida na literatura^(34)^. Atualmente, a região Norte do Brasil mostra níveis de pobreza significativamente mais elevados do que no resto do país^(35)^. Portanto, metas propostas com atenção especial para as populações mais pobres e em situação de vulnerabilidade e a garantia de que tenham acesso a serviços básicos, como o cuidado pré-natal, são fundamentais.

O ODS-3 infere assegurar uma vida saudável e promover o bem-estar para todos, em todas as idades. O ODS-5 menciona alcançar a igualdade de gênero e o empoderamento de todas as mulheres e meninas. Na região Norte, os indicadores como taxa de mortalidade materna e infantil, assim como outros, apresentam piores condições que no resto do país^(33)^ e, portanto, as metas relativas a esses objetivos estão diretamente relacionadas às condições de saúde na região Amazônica. Propostas como reduzir as mortes evitáveis de mães, recém-nascidos e crianças menores de cinco anos até 2030, assegurar consultas pré-natais, orientações sobre métodos contraceptivos, direitos sexuais e reprodutivos são fundamentais para uma vida saudável e o bem-estar das mulheres e meninas, sobretudo entre aquelas que se encontram em situação de maior vulnerabilidade, como as mulheres em situação de rua.

### Limitações do estudo

As limitações do estudo referem-se às entrevistas terem sido realizadas remotamente e à impossibilidade da observação dos atendimentos da equipe de CnaR, em especial do enfermeiro. Embora a coleta de dados no ambiente virtual possa oferecer riscos, foram tomadas medidas para a proteção, segurança e preservação dos direitos dos participantes da pesquisa^(36)^. O pesquisador principal realizou o *download* dos dados, apagando todos os registros em ambientes virtuais compartilhados. Salienta-se que a realização do estudo remoto possibilitou a inclusão de enfermeiros de diferentes estados da região Norte e o conhecimento de distintas realidades no cuidado pré-natal de gestantes em situação de rua.

### Contribuições para as áreas da enfermagem, saúde, ou políticas públicas

Este estudo evidenciou a importância da atuação do enfermeiro, enquanto membro da equipe CnaR, para a acessibilidade geográfica e socio-organizacional ao cuidado pré-natal às gestantes em situação de rua e estratégias de enfrentamento. A atuação do enfermeiro no cuidado pré-natal no CnaR contribui para a acessibilidade de gestantes em situação de rua ao cuidado pré-natal, sendo importante e sustentadora em favor da saúde do binômio. Também é importante para a execução de políticas públicas, contribuindo para a saúde e o bem-estar, redução da mortalidade materna e infantil, enfrentamento das desigualdades sociais e defesa de direitos humanos das mulheres enquanto elementos imprescindíveis para o alcance dos ODS. O cuidado de enfermagem no CnaR mostrou-se uma estratégia potente para o fortalecimento de vínculos, acolhimento e rastreamento das necessidades de saúde das mulheres que se encontram em situação de rua.

## CONSIDERAÇÕES FINAIS

O cuidado pré-natal pela equipe de enfermagem do CnaR é repleto de desafios, como pode-se observar com a ocorrência de captação tardia, descontinuidade do acompanhamento, mudanças de território e precariedade das condições de vida inerentes ao viver sem habitação. No entanto, a atuação do CnaR proporciona encontro com as gestantes *in loco* no território, propiciando a acessibilidade geográfica e socio-organizacional ao cuidado pré-natal, com acolhimento, construção de vínculos, educação em saúde e inserção na Rede de Atenção à Saúde. Nessa perspectiva, o enfermeiro do CnaR contribui para assegurar o direito à saúde das gestantes em situação de rua, colaborando para o alcance dos ODS.
